# Reduced Expression of *IL-1β* and *IL-18* Proinflammatory Interleukins Increases the Risk of Developing Cervical Cancer

**DOI:** 10.31557/APJCP.2019.20.9.2715

**Published:** 2019

**Authors:** Jose Anibal Matamoros, Maria Isabel Ferreira da Silva, Patrícia Muniz Mendes Freire de Moura, Maria da Conceição Gomes Leitão, Eliane Campos Coimbra

**Affiliations:** 1 *Laboratory of Molecular Biology of Viruses, Biological Sciences Institute, University of Pernambuco,*; 2 *Laboratory of Molecular Studies and Experimental Therapy, Department of Genetics, Biological Sciences Center, Federal University of Pernambuco, Recife, Brazil. *

**Keywords:** Cervical cancer, HPV, IL-18, IL-1β, biomarker

## Abstract

**Background:**

The objective of this study was to analyze the gene expression profile of the proinflammatory interleukins, *(IL-1β* and *IL-18*) in patients with premalignant lesions and cervical cancer.

**Methods:**

Total IL-1β and IL-18 mRNA was quantified by qPCR to obtain the expression data in cervical tissues. A total of 74 cervical biopsies were obtained from women undergoing a colposcopy. The samples were divided into: normal (19), low level lesions (LSIL) or NIC I (17), high level lesions (HSIL) or CIN II and CIN III (29) and cancer (9). The normal cervical tissue samples were included as controls. The OR and 95% CI were calculated for the determination of the risk of progression between each type of lesion and cancer using logistic regression.

**Results:**

The results showed that an increase in the risk of progression of pre-neoplastic lesions to cancer was between 2.5 and 2.08 times higher in women with lower *IL-1β* and *IL-18* expression, respectively.

**Conclusions:**

This study provided evidence that *IL-1β* and *IL-18* are potential biomarkers that can be explored in further studies for monitoring the evolution of pre-neoplastic lesions and avoiding overtreatment or undertreatment of the patients.

## Introduction

Cervical cancer ranks second in order of incidence in women in developing countries, with approximately 527,600 new cases per year (Torre et al., 2015). An annual increase in incidence of 2% is estimated in countries with a low human development index, where the mortality rate of new cases reaches 86% (Forman et al., 2012). 

Human papillomavirus (HPV) is the main cause of cervical cancer (zur Hausen, 2001) and it has been shown that infection and cancer progression depend on the type of HPV and the ability of the host immune system to control viral infection (Doorbar, 2006). In about 80% of cases, women have HPV infection at some point in their life (Moscicki, 2005). In addition, it is estimated that 10% of them develop chronic HPV infection which in 1% leads to cancer (Sasagawa et al., 2012). The development of cervical cancer usually takes about 10 to 20 years after HPV infection (Burd, 2003). Thus, this type of cancer is often preventable or treatable if detected early. However cervical cancer screening tests still have limited sensitivity (such as histopathology and cytology) (Saslow et al., 2012) as well as limited specificity (such as HPV DNA detection) (Ronco et al., 2006) to provide an accurate diagnosis and prognosis of CINs. Studies have shown that HPV adopts different strategies to evade the immune system (Miura et al., 2010). Several authors have demonstrated the ability of HPV to reduce the amount of INFγ, as well as to check the activation of NK cells and T cells with antiviral and antitumor activity (Sasagawa et al., 2012). Furthermore, HPV has been shown to have numerous mechanisms for altering the immune response that is mediated by inflammasomes (Sasagawa et al., 2012). On the other hand, it is known that the triggering of inflammasomes leads to the activation of interleukins (*IL-18* and *IL-1β*) (Dinarello, 2009). Both interleukins are activated by caspase-1 in response to the recognition of PAMPs or DAMPs, which is carried out by one of the four protein complexes that form the inflammasomes. Although they act through different mechanisms, both interleukins lead to an increase in the activation of inflammatory genes, T cell activity, NK cells, CD8 + T cells and the stabilized production of INFγ and TNFα (Kaplanski, 2018). Subsequently, *IL-1β* and *IL-18* trigger the activation of the adaptive immune response to viruses and tumor cells (Dinarello, 2009). In the light of this, the aim of this study was to study for the first time the changes in cervical tissue expression (normal, premalignant and malignant) of both interleukins in order to identify potential biomarkers for cervical carcinogenesis (Gravitt, 2011).

## Materials and Methods

*Description of samples*

**Figure 1:**
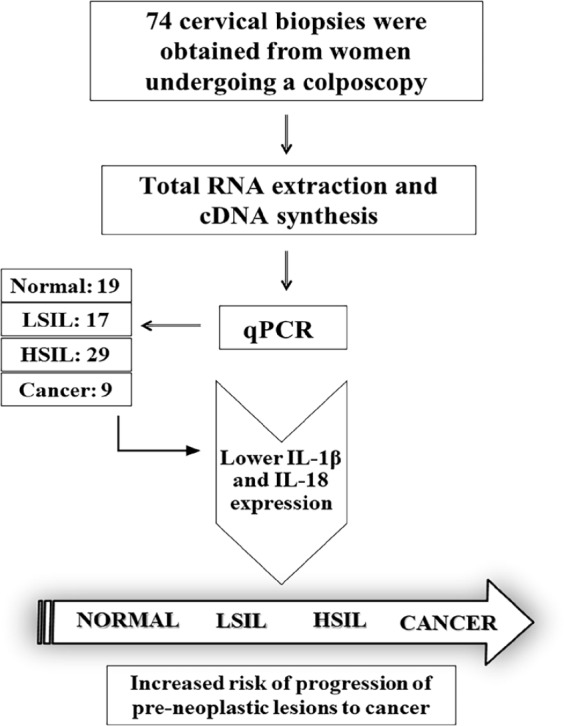
Flow Diagram of Study

The experiments were planned and carried out in accordance with the MIQE guidelines (Bustin et al., 2009). The biopsies of patients were collected at the Clinical Hospital of the Federal University of Pernambuco (UFPE), Brazil and Institute of Integral Medicine - Prof. Fernando Figueira (IMIP), Pernambuco, Brazil. A total of 74 cervical biopsies were obtained from women undergoing a colposcopy. The samples were divided into: normal (19), low level lesions (LSIL) or CIN I (17), high level lesions (HSIL) or CIN II and CIN III (29) and cancer (9) (Figure 1). The normal cervical tissue samples were included as controls. Women were excluded if they had the Human Immunodeficiency Virus (HIV) and/or suffered from medical conditions that significantly affected the immune response (owing to pregnancy, rheumatoid diseases, and other cancers), as well as those with an impaired understanding of the study. Fresh cervical biopsies were immediately preserved in RNAlater solution (Qiagen) and stored at -80°C. 

*Ethical approval*

All procedures in studies involving human participants were undertaken in accordance with the ethical standards of the institutional research committee and in compliance with the 1964 Helsinki Declaration and its later amendments or comparable ethical standards. The protocol for this study was reviewed and approved by the Research Ethics Committee of the Federal University of Pernambuco and Institute of Integral Medicine Prof. Fernando Figueira (protocol number: 03606212.7.0000.5208), and informed consent was obtained from all the individual participants included in the study.

*Total RNA extraction and cDNA synthesis*

The preserved samples were ground while still nitrogen-frozen and homogenized with 1 ml of Trizol (Invitrogen) for isolation of total RNA, in accordance with the manufacturer’s instructions. Total RNA was purified in a subsequent stage by means of the miRNA Absolutely RNA Kit (Agilent Technologies). The RNA quality was assessed by a NanoDrop 2000 Spectrophotometer (ThermoScientific, Wilmington, DE, USA) and 1% agarose gel electrophoresis (Bustin et al., 2009; Leitão et al., 2014; Rueda-Martínez et al., 2014). After this, 1 μg of RNA samples of a suitable quality (an OD260/280 from 1.8 to 2.1 and intact rRNA subunits - 28S and 18S) was used to generate the cDNA with the aid of a miScript II RT kit (Qiagen). A negative control RT reaction (no Reverse Transcriptase enzyme) was prepared for each sample to control genomic DNA contamination.

*Real-time quantitative polymerase chain reaction (qPCR)*

The Rotor Gene 6000 thermocycler (Qiagen) was used to run the qPCR reactions. The sequences of two primer pairs (*IL-1β* forward: 5’-AAATACCTGTGGCCT TGGGC-3‘, *IL-1β* reverse: 5’-TTTGGG ATCTAC ACT CTCCAGCT-3’, and *IL-18* forward: 5’-ATCGCT TCCTCTCGCAACAA-3‘, *IL-18* reverse: 5’-TCC AGGTTTTCATCATCTTCAGC-3’), were obtained through the studies of Nhu et al., (2010) and Niebler et al., (2013) respectively. The amplification efficiency for each primer pair was determined by a qPCR assay using the triplicates of a 10-fold dilution series (1:10, 1:100, 1:1,000, 1:10,000, 1:100,000) of normal cervical tissue cDNA as a template. The mean Ct values for each serial dilution were plotted against the logarithm of the cDNA dilution factor. The amplification efficiency for each primer pair was calculated by standard curve methods using the Efficiency  =  (10^(−1/slope)^-1)×100 formula. The melting curve was obtained to confirm the specificity of the primers. After the amplification efficiency had been checked, the quantification of cDNAs from the biopsies was conducted. The reaction conditions for the quantification of IL-1β mRNA were as follows: 15 min at 95°C (initial activation of HotStarTaq DNA Polymerase), followed by 40 cycles of 94°C for 15 seconds and 60°C for 60 seconds (combined); then the final extension at 72°C for 1 minute. The reaction conditions for the quantification of IL-18 mRNA were the same for IL-1β and only differed in the annealing and extension temperature step that was 62°C for 60 seconds (for 40 cycles). Reference genes that had been previously validated by the group of cervical tissues, were used to obtain mRNA expression levels (**26???**). In this way, the geometric mean of GAPDH and the ACTB reference genes, was used to calculate the relative expression of IL-1β and IL-18 mRNAs (Leitão et al., 2014). Every qPCR reaction was run in duplicate for each sample to minimize pipetting error (Nolan et al., 2006). Additionally, no template controls and no RT controls were added to detect contamination. 

**Table 1:** Risk For the Development of Cancer Based on the Expression of the *IL-1β *and *IL-18* Genes in Cervical Tissues

Condition of cervical tissue	N	Average	OR	Error	95% CI
Interleukin1 Beta					
Normal	19	0.36	1.85	0.56	(1.01-3.38)
LSIL	17	0.56	2.5	1.16	(1.00-6.23)
HSIL	29	0.85	1.29	0.24	(0.89-1.88)
Cancer	9	0.53			
Interleukin 18					
Normal	15	-4.91	1.6	0.56	(0.81-3.18)
LSIL	17	-4.6	2.65	1.35	(0.97-7.23)
HSIL	25	-6.44	2.08	0.69	(1.08-3.99)
Cancer	9	-5.19			

**Figure 2:**
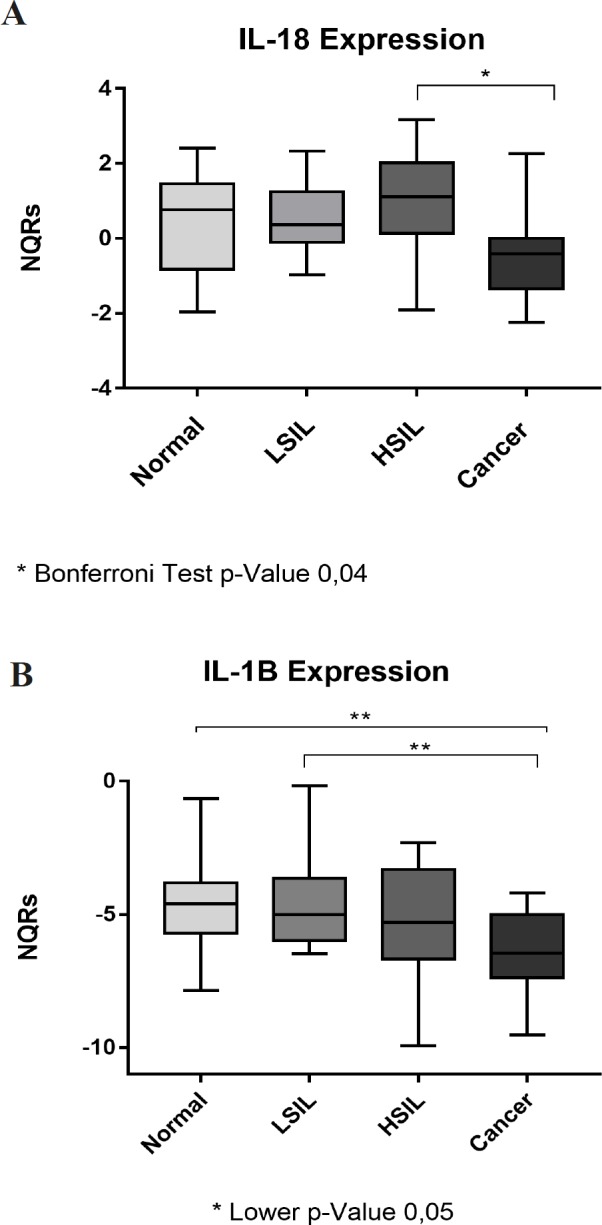
*IL-18* (A) and *IL-1β* (B) Gene Expression Levels in Cervical Carcinogenesis. In Figure A, a statistically significant difference can be seen in* IL-18* NQRs between HSIL and cancer resulting from the ANOVA test. Figure B shows a statistically significant difference between the averages of *IL-1β* NQRs, between the normal condition and cancer and LSIL and cancer groups using the t student test

*Statistical analysis and information processing*

Expression data processing was carried out in the Stata statistical software: Release 14.0(^©^Copyright 1996-2018 StataCorp LLC). Shapiro-Wilk test was carried out to determine the distribution of the data. The sample was standardized through logarithmic conversion of the *IL-18* NQRs (normalized relative quantities) and* IL-1β *NQRs (Hellemans et al., 2007). Then an ANOVA test was conducted to establish the link between the *IL-18* NQRs and the cervical tissue conditions. The Bonferroni test correction was used to determine which groups showed significant differences. In addition, the student t test was used to determine if there was an association between the NQRs of *IL-1β* and cervical tissue conditions, and to compare the mean averages with regard to the cancer group. The P value <0.05 was considered as statistically significant. In the case of the groups that did not have a statistical relationship, the error β was calculated with the aid of the OpenEpi3.01 statistical program. The odds ratio and 95% confidence intervals were calculated for a logistic regression to establish the risk of tumor progression in accordance with the *IL-18* and *IL-1β* NQRs.

## Results

The expression profile of *IL-18* and *IL1-β* and correlation with different stages of cervical carcinogenesis.

With regard to the* IL-18* expression profile in cervical tissues, the standardized relative quantities (NQRs) were 0.36 in normal samples; 0.56 in LSIL; 0.85 in HSIL and -0.43 in Cancer. There was a significant reduction of IL-18 expression in the HSIL samples (p <0.05) when compared with normal lesions; however, we did not find any difference between cancer and normal lesions, nor between cancer and LSIL, because error B in each of them was 0.73 and 0.54, respectively. These results can be seen in Figure 2A. Interestingly,* IL-1β* displayed a different expression pattern to that of *IL-18*. Figure 2B shows how the expression of this mRNA decreases in different types of lesions, even though a linear regression correlation was not established. The average expression was -4.91 for normal tissue; -4.6 for LSIL; -5.34 for HSILand -6.44 for cancer. A statistically significant decrease (p <0.001) was found between the average expression of normal tissue and cancer. Likewise, a significant decrease (p <0.001) of LSIL for cancer was demonstrated. Despite these results, it was not possible to establish a relationship between HSIL and cancer, because, owing to the sample size, there was a B error of 0.63.

*Determination of risk of progression of IL-1β and IL-18 between different stages of cervical carcinogenesis.*

Each cancer group was compared using logistic regression and odds ratio determination and confidence intervals to assess the risk of progression between each stage. In the case of *IL-18*, a significant (p <0.001) increase of 2.08 (CI% 95 1.06-3.99) was noted which shows there is a risk of the cancer progressing from HSIL if the *IL-18* expression decreases. However, no significant risk was found between normal conditions and cancer, nor between LSIL and cancer (Table 1). These results suggest that *IL-18 *could be used as a segment-based biomarker for patients classified with HSIL. The same test was used for *IL-1β* where an increased risk of 2.5 (95% CI 1-26.23) was calculated for progression from LSIL to cancer, when the *IL-1β* mRNA expression decreased. However, there was no change in the risk of clinical relevance between HSIL and Cancer. It should be noted that the reduction in *IL-1β* expression also represented an increased risk between the normal and cancerous groups.

## Discussion

The development of cervical cancer is characterized by permanent infection with high-risk HPV and local chronic inflammation that leads to the development of carcinogenesis (Piersma, 2011). A key feature of HPV-induced carcinogenesis, is that chronic inflammation is one of the main promoters of oncogenic pathway activation, and several studies have shown that the activation of *IL-1β* and* IL-18* proteins by inflammasome increases the progression of different types of cancer (Thi and Hong, 2017). These findings coincide in so far as the activation of inflammasomes creates inflammatory microenvironments conducive to tumorigenesis (Thi and Hong, 2017). On the other hand, viral replication must occur over a long period of time for the development of cervical cancer, which means that the HPV virus has developed different mechanisms to evade a host´s immune response (Sasagawa et al., 2012). 

There is sufficient evidence to confirm that the activation of *IL-18 *and *IL-1β* by inflammasome is a mechanism that can combine the progression and synergy between the innate and adaptive immune response system to produce competent cells with antiviral and antitumor activity (Gram et al., 2012). Given these discrepancies between the pro-oncogenic and antitumor activity caused by the activation of the *IL-18* and* IL-1β* proteins, the aim of this study was both to evaluate the changes in the mRNA expression of these interleukins in cervical tissue, and to identify the variations in profiles that could be linked to degrees of malignancy in cervical premalignant lesions and tumor progression.

Our results showed a significant reduction in* IL-18 *expression between high grade lesions (HSIL) and cancer. This reduced *IL-18* mRNA expression can increase the risk of developing cervical cancer. Contrary to the results obtained in this study, in the different viral infections caused by the following oncogenic viruses - Epstein Barr Virus (EBV) (van de Veerdonk et al., 2012), Hepatitis B Virus (HBV) and Hepatitis C Virus (HCV) (Sharma et al., 2009), - there was an increase in expression of *IL-18*. In addition, in the case of patients with HIV-1, a remarkable increase in *IL-18* expression was reported during the viral replication phase and progression of the infection (Stylianou et al., 2003). These differences suggest that there are mechanisms that reduce the expression of *IL-18* in the infection and progression of HPV-induced lesions. In uterine cervix cancer, there are some *IL-18* polymorphisms that have been linked to cancer progression (Tavares et al., 2016) and it has been found that a reduction of plasma IL-18 increases the risk of developing cervical cancer (Gening et al., 2014). Similar results were found in HeLa, Caski cells, monocytes and HPV-16 tumor associated macrophages (Cho et al., 2001; Lee et al., 2001). It is worth noting that HPV has been described as having strategies that reduce *IL-18* activity. Thus, it was demonstrated that the interaction of the E6 viral oncoprotein with *IL-18* increases the degradation of this interleukin via ubiquitin (Cho et al., 2001). As well as this, the E6 and E7 proteins inhibit *IL-18* activity by competitive binding to the *IL-18* receptor alpha chain, by block the activation of interferon-producing NK cells (Lee et al., 2001). In light of the results obtained from this study, it has been shown that there is a reduction in the local expression of the *IL-18* mRNA, which suggests that it is a new mechanism, which may lead to the persistence of HPV in the epithelium and consequent onset of cervical neoplasias and cancer.

Decreasing local *IL-18* can be regarded as a mechanism that HPV can use to evade the immune response, because *IL-18* is required to induce the activation of CD4+ T cells, CD8+ T cells, and NK cells (Kaplanski, 2018). On the other hand, *IL-18*, in synergy with *IL-12*, stabilizes the production of INFγ, which results in more potent cytotoxic activity of T cells. In addition, there is a reduced activity and immune capacity in the *IL-18* -/- NK cells, and it has been reported that *IL-18*-deficient mice develop a greater inflammatory response and cancerous lesions in the intestinal epithelium (Salcedo et al., 2010). Other studies have found that *IL-18* exerts a control to maintain homeostasis between inflammation and repair of the lamina propria that lies beneath the epithelium (Dupaul-Chicoine et al., 2010). It is worth pointing out that, in osteosarcoma, increased *IL-18* expression can be attributed to an increase in tumor resistance derived from the infiltration of suppressor cells of myeloid origin (Guan et al., 2017). However, in melanoma it has been observed that *IL-18* enhances the antitumor response by inducing tumor-infiltrating CD8+ T lymphocytes (Kunert et al., 2017). These results show that *IL-18 *plays an important role in the control of tumor cells.

The expression profile of *IL-1β* in the different tissues affected by cervical conditions was similar to that of* IL-18*, with a significant decrease in the mRNA expression levels between normal controls and cancer, as well as between* LSIL* and cancer. Interestingly, we did not observe a difference between HSIL and cancer. In other analyses, increased *IL-1β* expression was related to a greater risk of developing different types of cancer, because it causes tumorigenesis, angiogenesis and metastasis through different mechanisms (Apte and Voronov, 2008). *IL-1β* was described as an interleukin which creates a microenvironment conducive to the development of secondary cancer, together with increased fibroblast activity, stabilization in the transcription of the *MMP-1* gene and collagens (Müerköster et al., 2004). Furthermore, *IL-1β* facilitates cell migration by stimulating the synthesis of HIF-1α under non-hypoxic conditions (Filippi et al., 2015). It also causes angiogenesis by increasing CxCL8 / CXCR1 (Filippi et al., 2015), VEGF (Voronov et al., 2003), *EGFR* and *IL-8 *(Lee et al., 2015), as well as secondary tumorigenesis with a greater production of ROS, COX2, NADPH oxidase and reduction of 15-PGDH (Arima et al., 2017). Some authors attribute the rise in *IL-1β* plasma to an increased risk of cervical cancer in women with polymorphisms (Qian et al., 2010). Likewise, a greater concentration of* IL-1β* in the vaginal mucosa was reported in women with high grade cervical lesions (Iwata et al., 2015). 

The different results in the *IL-1β* relationship obtained from the development of different types of cancer, leads us to believe that the reduction in *IL-1β *expression is a protective factor for the development of cancer. However, the results of this study suggest that in cervical cancer this does not appear to be the rule. Other authors have reported a decrease in *IL-1β* expression in cells derived from cervical carcinoma, SiHa, C4-1, and HPV-infected keratinocytes (Merrick et al., 1996). Although there is a discrepancy between the expression behavior in tissue and cell cultures, it is known that HPV has mechanisms to block *IL-1β* activity. Niebler (2013) recently described how HPV can control *IL-1β* secretion to evade the response caused by the immune system through posttranslational modifications orchestrated by the formation of E3-ubuquitin ligase E6-AP ligand and p53 trimer, and leading to the degradation of proIL-1β in cells infected with HPV-16. Likewise, non-differentiated keratinocytes that are positive for HPV-16 and HPV-18, have been shown to reduce *IL-1β *expression through mechanisms mediated by CDKN2A (Karim et al., 2011). Subsequently, HeLa cells, stimulated by* IL-1β*, have been shown to decrease the ability to metabolize xenobiotics, which are not beneficial for viral survival (Mosaffa et al., 2012).

Overall, the control of human papillomavirus infection depends on having a suitable immune response, and requires the activation of *IL-1β* and *IL-18*, through inflammasomes, to stimulate a coordinated and effective response between the innate and adaptive immune systems. However, HPV is able to block the expression of some of the components of inflammasomes (Karim et al., 2011). In addition, HPV has developed different mechanisms that can block the expression and activity of *IL-1β* and *IL-18* proteins, and it has been reported that in cervical carcinogenesis, there is an increase in the expression of *IL18BP* receptors (Richards et al., 2014) and *IL-1Rα* (Fujiwaki et al., 2003), which act as antagonists of *IL-18* and* IL-1β*, respectively. This might be significant because blocking the expression of both interleukins could be one of the viral strategies for evading the immune response and hence causing chronic infection and cancer (van de Veerdonk et al., 2011). Moreover, blocking the activity of both interleukins affects the formation and activation of mature NK cells and components for viral control, and reduces their capacity to recruit neutrophils with anti-tumor capacity at the lesion site. In addition, it reduces the cytotoxic activity of CD8+ T cells, as well as the M1 macrophages with antitumor capacity, and finally, leads to a reduction in the local production of INFγ required for viral control (Dinarello, 1996; van de Veerdonk et al., 2011).

With regard to the clinical diagnosis of cancer, these results can be used as a tool to monitor the evolution of pre-neoplastic lesions, since the increase in the risk of progression of pre-neoplastic lesions to cancer was found to be 2.5 and 2.08 times higher in women with lower *IL-1β* and *IL-18* expression, respectively. Thus, this study was able to obtain the expression profile of* IL-18* and *IL-1β* interleukins in cervical tissues for the first time, and demonstrate that the reduction in *IL-1β* expression is linked to an increased risk between normal and cancer patients and that the reduction in IL-18 expression in HSIL patients can be used as a follow-up biomarker. In addition, these results provide new information on the behavior of HPV-induced carcinogenesis and form the basis of potential therapeutic targets for the control of human papillomavirus infection. On the other hand, there are as yet no effective tests or biomarkers that can show when a pre-neoplastic lesion will progress to a more advanced state. For this reason, the use of biomarkers that enable the risk of progression to be monitored represent a useful tool for the segmentation and tracking of cervical cancer.
